# Do modern climatic niches distinguish extinct and extant plant genera in New Zealand?

**DOI:** 10.1002/ece3.70133

**Published:** 2024-09-03

**Authors:** Nora Schlenker, William G. Lee, Tammo Reichgelt, Ralf Ohlemüller

**Affiliations:** ^1^ Department of Geography University of Wisconsin‐Madison Madison Wisconsin USA; ^2^ Manaaki Whenua – Landcare Research Dunedin New Zealand; ^3^ Department of Earth Sciences University of Connecticut Storrs Connecticut USA; ^4^ School of Geography University of Otago Dunedin New Zealand

**Keywords:** Australia, Cenozoic, climate, cooling, extinction, flora, New Zealand, niche, Schoener's *D*

## Abstract

Past climate changes have had large impacts on modern ecological patterns. Understanding if legacies are distinguishable in the climatic niches of extant and locally extinct taxa can provide insight into the importance of climate in extinction events. To better understand mid‐ to late‐Cenozoic New Zealand plant extinctions, which are often attributed to Cenozoic climate cooling, we identify 13 con‐familial extinct and extant New Zealand genus pairs, which have modern distributions in Australia. Using climatic niches derived from current geographic distributions in Australia, we compared (i) total niche breadth, (ii) niche overlap, and (iii) individual climate parameters, to investigate potential climate drivers of intrafamilial extinction and persistence patterns in New Zealand. A majority of New Zealand extinct genera (9 out of 13 pairs) do not indicate climate niche legacies consistent with susceptibility to extinction from changing climates, while the remaining four extinct/extant pairs show slight climatic niche legacies. Three extinct genera have warmer niches than their extant counterpart, which is consistent with extinction reflecting intolerance of cooling Cenozoic climates. The other genus pair with a climatic niche legacy has an extinct genus that is distinguished by a niche with smaller precipitation seasonality than its extant counterpart, suggesting that climate metrics other than temperature may also be important extinction drivers in some taxa. Our results show that the mechanisms of Cenozoic extinctions of New Zealand genera are likely more complex than taxa reaching environmental tolerances due to cooling climates. Comparisons of current climatic niches between extant and extinct sister taxa can provide useful insights into large‐scale, long‐term climatic legacies but more analyses, including trait and phylogeographic patterns, would lead to additional insights into alternative pathways of extinction.

## Graphical abstract

We aim to better understand New Zealand plant extinctions, often attributed to Cenozoic climate cooling, through determining if legacies of past extinction events are detectable in the climatic niches of con‐familial genera in the modern Australian floral assemblage. We find that the mechanisms of Cenozoic extinctions of New Zealand genera may be more complex than taxa reaching environmental tolerances from cooling climates. Comparisons of current climate niches between extant and extinct sister taxa can provide useful insights into large‐scale, long‐term climatic legacies.

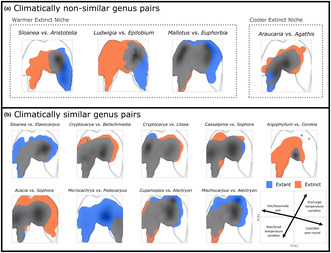

## 1 INTRODUCTION

Climate is a key determinant of the large‐scale spatial distribution of plant species and ecosystems (McGill, 2010; Willis & Whittaker, 2002). Multiple lines of evidence show changes in climate as a cause of recent and paleontological floral extinctions (Cahill et al., 2013; McElwain & Punyasena, 2007) and as a predicted cause for future extinctions under anthropogenic climate change (Thomas et al., 2004; Urban, 2015). It is important to understand how species niches have responded to past climate changes to understand the susceptibility of flora to climate‐mediated extinctions. Plant niches can be described as the set of environmental conditions in which a taxa's population can persist (Hutchinson, 1957). The fundamental niche includes all possible factors required for a species to survive, while the realized climatic niche is the estimation of the niche based on the current climate‐distribution relationship and is limited by the breadth of current climate space (Jackson & Overpeck, 2000). Therefore, the realized climatic niche of a species can change through time and space as areas of the fundamental niche appear or disappear based on the available climate within geographic space (Jackson & Overpeck, 2000), while at some taxonomic levels (i.e. within genera), the fundamental climatic niche can evolve through speciation (Emery et al., 2012; Lu et al., 2023).

Plants can respond to climate changes in three ways: move, adapt, or go extinct. Plant movement in response to climate change has been extensively studied. Plant species may respond to climate changes through geographic migration to track suitable climates thus retaining their climatic niches (i.e. niche conservatism). For example, in response to deglacial warming, many taxa in Eastern North America migrated northward to follow cooler climates (Davis, 1981; Davis & Shaw, 2001; Prentice et al., 1991) and have maintained their climatic niches through the last 18,000 years (Wang et al., 2023). A similar pattern can be seen in response to cooling climates between the last interglacial and the last glacial maximum in flowering stonecrops (*Rhodiola* spp.) on the Qinghai‐Tibetan Plateau which migrated to lower elevations and latitudes to track warmer climates (You et al., 2018). On longer time scales, the genus *Laurus* has had a stable climatic niche over the last 3 million years, as the reconstructed paleoclimate of *Laurus*' paleogeographic range matches its modern climatic niche (Rodríguez‐Sánchez & Arroyo, 2008). This demonstrates that through the three climate shifts analyzed – the middle Pliocene, the last glacial maximum, and modern – *Laurus* climatic tolerances remained stable, and individuals moved to track suitable climates (Rodríguez‐Sánchez & Arroyo, 2008). Niche conservation has been invoked for many palaeoecological studies that use modern climatic tolerances of taxa as analogs to estimate past climates based on fossil records (Chevalier et al., 2020; Prebble et al., 2017; Thompson et al., 2012).

Adaptation in response to climate change can also occur in plant taxa where plants evolve to survive in new climates as observed in changes to the taxon's climatic niche, i.e. niche divergence. Paired phylogenetic and niche studies can track niche divergence through speciation events (Lu et al., 2023). For example, the niches of species within the *Chrysanthemum zawadskii* complex have diverged since the Pliocene in response to range expansion (Lu et al., 2023). There is evidence for family and genus level niche conservatism over large spatial, temporal, and taxonomic scales but with niche divergence at the species level. For example, most genera within the family Cyatheaceae, scaly tree ferns, show global niche conservatism, generally occupying areas that are warm, frost‐free, and have consistent rainfall (Bystriakova et al., 2011). However, geographic isolation and phylogenetic dynamics contribute to Cyatheaceae species‐level niche divergence (Bystriakova et al., 2011). Modeling efforts have also shown that the degree of speciation and niche divergence can impact species ability to survive climatic changes (Aguilée et al., 2016; Rangel et al., 2018). Over shorter time periods niche divergence has also been witnessed within species that have been introduced to new geographic areas, likely due to competitive release or expanded availability of favorable climates (Guisan et al., 2014). However, these niche changes exhibit interspecies variability, with niche conservatism observed in some and niche divergence in others (Pearman, Guisan, et al., 2008). Niche divergence is also sensitive to spatial scale, being more common at smaller, habitat scales, while niche conservatism appears more prominent at larger climatic scales (Emery et al., 2012; Silvertown et al., 2005). This suggests niche divergence may result from competition and local adaptation, but that these dynamics are not distinguishable at larger spatial scales (Emery et al., 2012, Silvertown et al., 2005).

Climate mediated extinction events in plant taxa are less well understood than migrations and adaptations. For example, of the five global mass extinction events, none coincide with mass extinctions of plant taxa (Wing, 2004). However, regional extinctions are more prevalent, and are often associated with major abiotic events (e.g. Carvalho et al., 2021; Jaramillo et al., 2010). A sister genera analysis of Pliocene‐Pleistocene floral extinctions of European temperate tree taxa with high levels of niche conservation shows that extinct genera typically occurred in warmer areas than widespread genera, suggesting that glacial cycles in combination with migration barriers caused warm‐adapted genera to go extinct while more cold‐adapted taxa persisted (Svenning, 2003).

Past climate changes have resulted in many legacies in the species composition and spatial distributions of ecosystems today (Svenning et al., 2015). To further understand how past climate changes may have interacted with extinction events we determine if legacies of past extinction events can be distinguished within taxa climatic niches. We define niche legacies as a substantial difference in the climatic niches of closely related extinct and extant taxa in the modern assemblage which could be hypothesized to have played a determining role in an extinction event. Large climate legacies would suggest that differences in climate tolerances made the extinct taxa more susceptible to extinction from past climate changes than their extant counterparts. The flora of New Zealand gives us a rare opportunity to test if taxa retain climatic niche legacies of extinction due to its exemplary Cenozoic floral extinction record, and because many genera that went extinct in New Zealand are still extant in the modern Australian flora (Lee et al., 2001).

The Cenozoic was a time of substantial climatic, geologic, and ecological change in New Zealand. Consistent with global long‐term trends of cooling climates (e.g. Westerhold et al., 2020), New Zealand lost much of the tropical and subtropical climates that existed during early periods of the Cenozoic (Conran, Bannister, et al., 2016; Crouch et al., 2020; Kennedy et al., 2002; Pancost et al., 2013), transforming into the largely temperate climates seen today (Nelson & Cooke, 2001; Prebble et al., 2017). In addition to climate changes, New Zealand underwent geological changes as terrain expanded into the alpine bioclimatic zone (Prebble et al., 2021; Winkworth et al., 2005). Although the current New Zealand flora has a high level of species endemism, there is low endemism at the genus and family level due to common Gondwanan ancestry, either through persistence since separation or later dispersal from Australia and elsewhere (McGlone et al., 2001; Winkworth et al., 2002). Therefore, the current genus‐level composition of the New Zealand flora is a result of a shared Gondwanan history and subsequent genus‐level extinctions throughout the Cenozoic. The many genera that went locally extinct in New Zealand during the Cenozoic has largely been attributed to climatic cooling but few comparative studies of the environmental tolerance of extinct and extant genera have been undertaken (Lee et al., 2001).

In New Zealand, Early Cenozoic climates were tropical to subtropical, with mean annual temperatures ranging from 20 to 23°C in the Eocene (56–34 mya, Conran, Bannister, et al., 2016; Pancost et al., 2013) but cooled to approximately 18°C in the Oligocene (34–23 mya), before increasing in the middle of the Miocene (23–5 mya), to between 18 and 20°C, accompanied by increasing temperature seasonality (Prebble et al., 2017; Reichgelt et al., 2019). During the Oligocene and Miocene, marine transgression submerged as much as 80% of the New Zealand land area (Cooper & Cooper, 1995; Lee et al., 2001). Mountain building, relative plate motion changes, and global sea level drop then increased land area resulting in the creation of cooler high‐elevation climates by the late Miocene (Barth et al., 2014; Cooper & Cooper, 1995; Lee et al., 2001; McGlone et al., 2001). The Pliocene (5–2.6 mya) and Pleistocene (2.6 mya–10,000 years ago) are marked by a sharp decrease in mean annual temperature, to 15°C and below, and increasing seasonality (Prebble et al., 2017). Average annual precipitation also increased through the Pliocene and Pleistocene, exceeding 2500 mm/year (Prebble et al., 2017). As Cenozoic climates changed in New Zealand, climate tracking may have become impossible for species as tropical and subtropical climates became geographically limited and eventually disappeared (Lee et al., 2001). Since there was no northward landmass for New Zealand genera favoring subtropical climates to migrate to, the available options were to either adapt to changing climates or go extinct.

The New Zealand Cenozoic fossil record is exemplary, including numerous sedimentary types (coal measures, lacustrine deposits) that preserve pollen, leaves, flowers, and wood of many taxa (e.g., Lee et al., 2001, 2016). A notable feature of this record is the late Cenozoic extinction of many groups that currently remain in Australia (Lee et al., 2001; Prebble et al., 2021). New Zealand and Australia share an early Cenozoic biota related to their common Gondwanan ancestry but have different climatic and extinction histories since separation in the late Cretaceous (Strogen et al., 2023), ~80 million years ago (MYA). Since separating from Gondwana, Australia maintained a larger subaerial landmass (>7 million km^2^) spanning a broad latitudinal range (currently 10–43° S), while New Zealand, in contrast, has less landmass (<0.3 million km^2^) and encompasses a narrower latitudinal range (currently 35–47° S). The persistence of many extinct New Zealand genera in Australia has been interpreted to reflect the consistent representation of tropical and subtropical environments throughout the Cenozoic (Lee et al., 2001).

**FIGURE 1:**
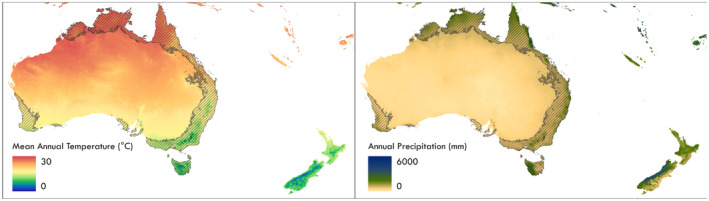
Mean annual temperature and annual precipitation of modern Australia from WorldClim Version 2 (Fick & Hijmans, 2017). Climatic niches are computed from the temperate to tropical portions of Australia (hashed area), determined by the Köppen–Geiger climate zones (Beck et al., 2018).

The temperatures and precipitation experienced in New Zealand throughout the Cenozoic (Table 1, subtropical to temperate) also correspond with a substantial portion of the current Australian climate space, where many genera once in New Zealand now reside, mostly concentrated along the Australian east coast (Figure 1). This provides an opportunity to understand the climatic niche legacies of past New Zealand extinctions because we can directly compare climatic niches of extinct and extant taxa controlling for the available climate space. Understanding these legacies can help determine why and how some genera remained in New Zealand while others became extinct.

**TABLE 1:** Past climate estimates of New Zealand for mean annual temperature and annual precipitation from Conran, Bannister, et al. (2016), Mildenhall (1980), Prebble et al. (2017), and Reichgelt et al. (2019).

Cenozoic period	Temperature	Precipitation
Eocene	20–23°C	1200–8000 mm
Oligocene to Early Miocene	18–20°C	1000–2500 mm
Middle Miocene to Pliocene	15–18°C	1000–3000 mm
Pleistocene	<15°C	1600–5000 mm

**FIGURE 2:**
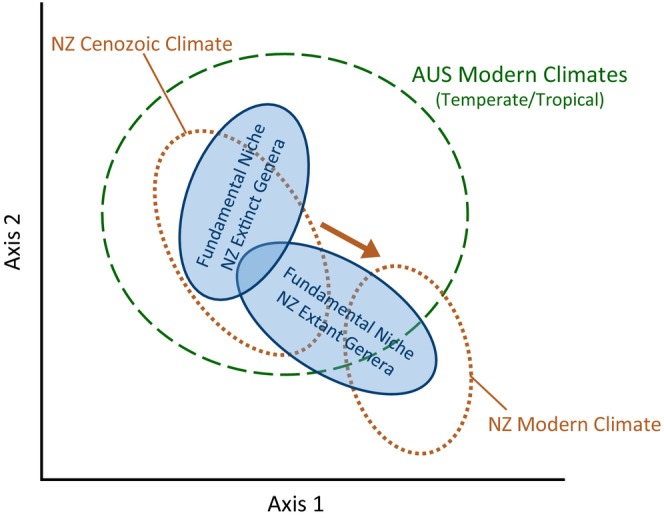
Conceptual model of the link between the changing New Zealand climate space through time, the fundamental niches of extinct and extant New Zealand taxa, and the modern Australian climate space. As the New Zealand climate space shifts (orange dashed) it may result in New Zealand no longer containing climate within the fundamental niche of some taxa resulting in extinction of that taxa (NZ extinct taxa). Other taxa fundamental niches may still overlap with the modern New Zealand climate allowing for the species to survive the New Zealand climate shift (NZ extant taxa). To understand differences in past niche dynamics these species can be observed in the modern Australian climate.

To test whether New Zealand climate changes influenced the extinction of genera in New Zealand, we compare the climatic niches of extant and extinct New Zealand genera based on their modern Australian distribution (Figure 2). We use the current climate distributions of genera to understand if modern realized niches show a legacy of climatic constraints that may have played a role in the extinction of genera from New Zealand. Specifically, this study looks to see if New Zealand extinct plant genera occupy different climatic niches than closely related New Zealand extant plant genera in the temperate and tropical portions of Australia which represent the full breadth of climates experienced by New Zealand during the Cenozoic (Figure 2). Niche conservatism over long time periods has been observed in other taxa (Huntley et al., 1989; Svenning, 2003) and using climate niches of modern taxa to estimate paleoclimates has been employed in many studies (Chevalier et al., 2020; Prebble et al., 2017; Reichgelt et al., 2015; Utescher et al., 2000).

We hypothesize that as the New Zealand climate cooled during the Miocene, Pliocene, and Pleistocene, the available climates in New Zealand moved outside of the fundamental niches of those species that went extinct in New Zealand (Figure 2). Because Cenozoic New Zealand climates are contained within modern‐day tropical and temperate zones of Australia (Table 1, Figure 1), we expect that the modern Australian realized niches of extinct New Zealand genera will be warmer than extant New Zealand genera, indicating that the loss of warm climates contributed to the extinction of those genera in New Zealand. If climate legacies of Cenozoic extinction events are present in the modern assemblage, then paired extinct and extant taxa will have substantially different climatic niches. To identify potential climatic correlates of extinctions of taxa in New Zealand we (i) quantify differences in total niche breadth, (ii) analyze similarities of climatic niches, and (iii) compare individual climate parameters between extinct and extant New Zealand genera in Australian climate space.

## 2 METHODS

### 2.1 Taxa selection and data

Con‐familial focal genera were selected to represent families that are present in Australia and have at least one genus that is extant in New Zealand and one that is only represented in the fossil record in New Zealand (i.e. Figure 2). This process resulted in the selection of 13 genus pairs across nine families. Global occurrence data was downloaded from the Global Biodiversity Information Facility (GBIF, 2023) for all nine families and filtered to include focal genera and distribution points in Australia (Appendix 1). Only data based on living or herbarium specimens (fossil specimens were not used) with a geographic location with uncertainty of less than half the width of a grid cell from the climate data (2250 m) were used.

**TABLE 2:** Climatic niche similarity between a New Zealand‐extinct genus and a related genus from the same family that is extant in New Zealand and Australia for nine families.

Family	Pair	Schoener's *D*	Niche similarity test	Niche difference test
Araucariaceae	***Araucaria* vs. *Agathis***	**0.33**	**–**	**–**
Argophyllaceae	*Argophyllum* vs. *Corokia*	0.37	‡	–
Elaeocarpaceae	*Sloanea* vs. *Elaeocarpus*	0.75	‡	–
	***Sloanea* vs. *Aristotelia***	**0.09**	**–**	**–**
Euphorbiaceae	***Mallotus* vs. *Euphorbia***	**0.44**	**–**	**–**
Fabaceae	*Acacia* vs. *Sophora*	0.32	†	–
	*Caesalpinia* vs. *Sophora*	0.72	‡	–
Lauraceae	*Cryptocarya* vs. *Beilschmiedia*	0.83	‡	–
	*Cryptocarya* vs. *Litsea*	0.85	‡	–
Onagraceae	***Ludwigia* vs. *Epilobium***	**0.06**	**–**	**–**
Podocarpaceae	*Microcachrys* vs. *Podocarpus*	0.24	†	–
Sapindaceae	*Cupaniopsis* vs. *Alectryon*	0.64	‡	–
	*Mischocarpus* vs. *Alectryon*	0.52	†	–

*Note*: Climatic niche similarity is based on the realized climatic niche of the two genera derived from their current Australian temperate and tropical distributions. Schoener's *D* values range from 0 (no overlap) to, 1 = full overlap = identical climatic niches and niche similarity and niche difference tests calculates a significance value for one genus while using a null model for the other genus, therefore each pair has two results (*p*‐values adjusted for multiple comparisons, – = both directions >.05, † = one direction <.05, ‡ = both directions <.05). Genus pairs that are non‐similar in at least one test are indicated in bold. Pairs are shown as New Zealand extinct vs. New Zealand extant genera.

### 2.2 Environmental space and climatic niche data

Using con‐familial genera of regionally extinct species attempts to control for the evolutionary changes to climatic niches that may occur over millions of years due to niche conservatism within many plant families that preserve genus‐climate relationships through time (Bystriakova et al., 2011; Svenning, 2003). Due to the millions of years involved, it is possible that these genera have adapted through time to novel climates in Australia, especially dryland ecosystems. We therefore restricted this study only to the temperate, sub‐tropical, and tropical portions of the Australian continent (Figure 1, hashed area). Here we compare the climatic niches of con‐familial pairs with one genus that is extinct in New Zealand and one genus that is extant in New Zealand that are both present in the tropical and temperate portions of Australia (Figure 2), which is a proxy for Cenozoic New Zealand climates (Table 1). Climatic niches for each taxon were quantified using their current distribution in temperate and tropical Australia. The areas that qualify as temperate and tropical Australia were determined by the Köppen‐Geiger climate zones (Beck et al., 2018) that are broadly consistent with past New Zealand climates (Table 1).

To account for geographic bias in GBIF data due to the clustering of points in cities or along major roads, the data was downscaled to match the resolution of the climate grid, 2.5 arc‐min, so that each grid cell associated with genera presence has the same weight in niche calculations. Some GBIF species occurrence points (less than 1 percent) did not overlap with the climate grid due to the climate grid's lower precision along coastlines and waterways and were excluded. After downscaling over 457,000 occurrence points remained for all nine plant families (Appendix 1).

The climate grid contains all 19 climate variables from WorldClim Version 2 at a spatial resolution of 2.5 arc‐min (approximately 4.5 km^2^) which are commonly used for niche modeling (Fick & Hijmans, 2017; Warren et al., 2008). Bioclimate variables included are annual mean temperature (bio 1), mean diurnal range (bio 2), isothermality (bio 3), temperature seasonality (bio 4), the maximum temperature of the warmest month (bio 5), minimum temperature of the coldest month (bio 6), temperature annual range (bio 7), mean temperature of the wettest and driest quarter (bio 8 and 9), mean temperature of warmest and coldest quarter (bio 10 and 11), annual precipitation (bio 12), precipitation of wettest and driest month (bio 13 and 14), precipitation seasonality (bio 15), precipitation of wettest and driest quarter (bio 16 and 17), and precipitation of warmest and coldest quarter (bio 18 and 19).

### 2.3 Niche overlap statistics

Niche overlap was calculated between genus pairs based on climatic niches in Australia with the aim of finding differences that could identify climate parameters responsible for the extinction of genera in New Zealand. The climatic niches of genera were determined using climate data associated with the distribution of each genus and performing a principal component analysis (PCA) to analyze climatic niches in two dimensions. The PCA was completed in R version 4.2.2 (R Core Team, 2022) using the package *ade4* version 1.7‐20 (Dray & Dufour, 2007) and the package *ecospat* (Di Cola et al., 2017). The method is consistent with calculating the most accurate measure of climatic niche overlap as described in Broennimann et al. (2012).

#### 2.3.1 Schoener's *D*

The Schoener's *D* metric (Schoener, 1968) was used to quantify the similarities and differences of the climatic niche of paired taxa. The metric calculates the amount of overlap between niche accounting for the frequency of occurrence for each input variable for both species, respectively. The Schoener's *D* metric is a value between 0 and 1 representing no niche overlap to complete niche overlap, respectively (Schoener, 1968). Schoener's *D* is typically used for niche comparison of microhabitat or diet and may not be appropriate for all environmental niche models (Warren et al., 2008). However, when compared against results from the Hellinger distance, no significant difference was observed (Warren et al., 2008). Calculation of Schoener's *D* first calculates an occurrence density grid for each genus using inputs from the first two PCA axes for each genus and the full available environmental space. Schoener's *D* overlap is then calculated based on the genera density grids while correcting for the density of the availability of climates within the study area (Di Cola et al., 2017). Schoener's *D* was completed in R version 4.2.2 (R Core Team, 2019) using the package *ecospat* version 3.4 (Di Cola et al., 2017).

#### 2.3.2 Niche similarity

The statistical significance of Schoener's *D* was determined following the methods of Warren et al. (2008) which compare the climatic niche of one taxon to null model that incorporates differences in the available climate space between ranges to determine if taxa are more similar than random. This is achieved by calculating the niche overlap using the actual, observed niche of one genus and comparing a niche that is randomly generated from the available climate space of this first genus, in this case the available climate space is the same for both species (Broennimann et al., 2012; Warren et al., 2008). A histogram of the 1000 null model generated Schoener's *D* values is then compared to the actual Schoener's *D* value using a two‐tailed test on the null distribution model to determine if the actual Schoener's *D* is greater than the 95% confidence interval's upper value, which tests for niche similarity, or lower value, which tests for niche difference test (Broennimann et al., 2012; Warren et al., 2008). This calculation is directional, with each pair having two estimates for niche similarity and niche difference. Genus pairs with at least one direction with *p* < .05 in the similarity test were considered to have similar climatic niches and those with at least one direction with *p* < .05 in the difference test were considered to have different climatic niches. Calculation of niche similarity was competed in R version 4.2.2 (R Core Team, 2019) using the package *ecospat* version 3.4 (Di Cola et al., 2017).

#### 2.3.3 Kernel density

In addition to analyses of overlap in multidimensional climatic niche space, climate overlap of each genus pair was analyzed separately for all climate variables using kernel density. Climatic distributions of genera are not normally distributed; therefore, the non‐parametric kernel density estimation (KDE) was used. To estimate overlap, KDE estimations were calculated for each genus and climate variable individually, these KDE estimates were then compared within the genera‐pairs and the percent overlap was computed. The KDE calculations were performed in R (R Core Team, 2019) using the package *overlapping* version 2.1 (Pastore, 2018).

### 2.4 Niche volume

Niche volume was calculated as the overlap between each individual genus climatic niche and the temperate and tropical Australian climate space. This was completed in two ways: (i) using Schoener's *D* as described above, and (ii) using the percent overlap in a multidimensional kernel density estimation. The Schoener's *D* niche volume and overlap value was generated for each genus with values between 0 and 1 representing none to full overlap between each genus and the temperate and tropical Australian climate space. Schoener's *D* was completed in R version 4.2.2 (R Core Team, 2019) using the package *ecospat* version 3.4 (Di Cola et al., 2017). The kernel density estimation of niche volume creates a two‐dimensional grid (PCA1 and PCA2) of kernel density and delimits the 95th quantile area for the temperate and tropical Australian climate space and each individual genus niche. The niche volume is then calculated as the percent of the temperate and tropical Australian climate space that is occupied by each genus. Kernel density niche volume analysis was completed in R version 4.2.2 (R Core Team, 2019) using the package *ks* version 1.14.1 (Duong, 2023).

## 3 RESULTS

**FIGURE 3:**
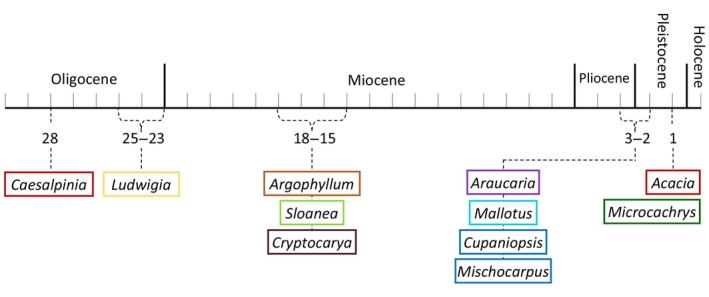
Presumed date of extinction from New Zealand for each focal genus and full list of extinct and extant genera. Extinction dates are based on the youngest known fossil record in New Zealand. Dates displayed in millions of years ago (MYA) and genera are color coded by family; for references see Appendix 1.

The extinction dates of genera in New Zealand are based on the youngest known fossils. All extinctions occurred within the Cenozoic (Figure 3). *Caesalpinia* had the earliest extinction, during the Oligocene approximately 28 million years ago (MYA), and *Acacia* and *Microcachrys* were the most recent extinctions, occurring approximately 1 MYA in the Pleistocene (Figure 3, Appendix 2).

**FIGURE 4:**
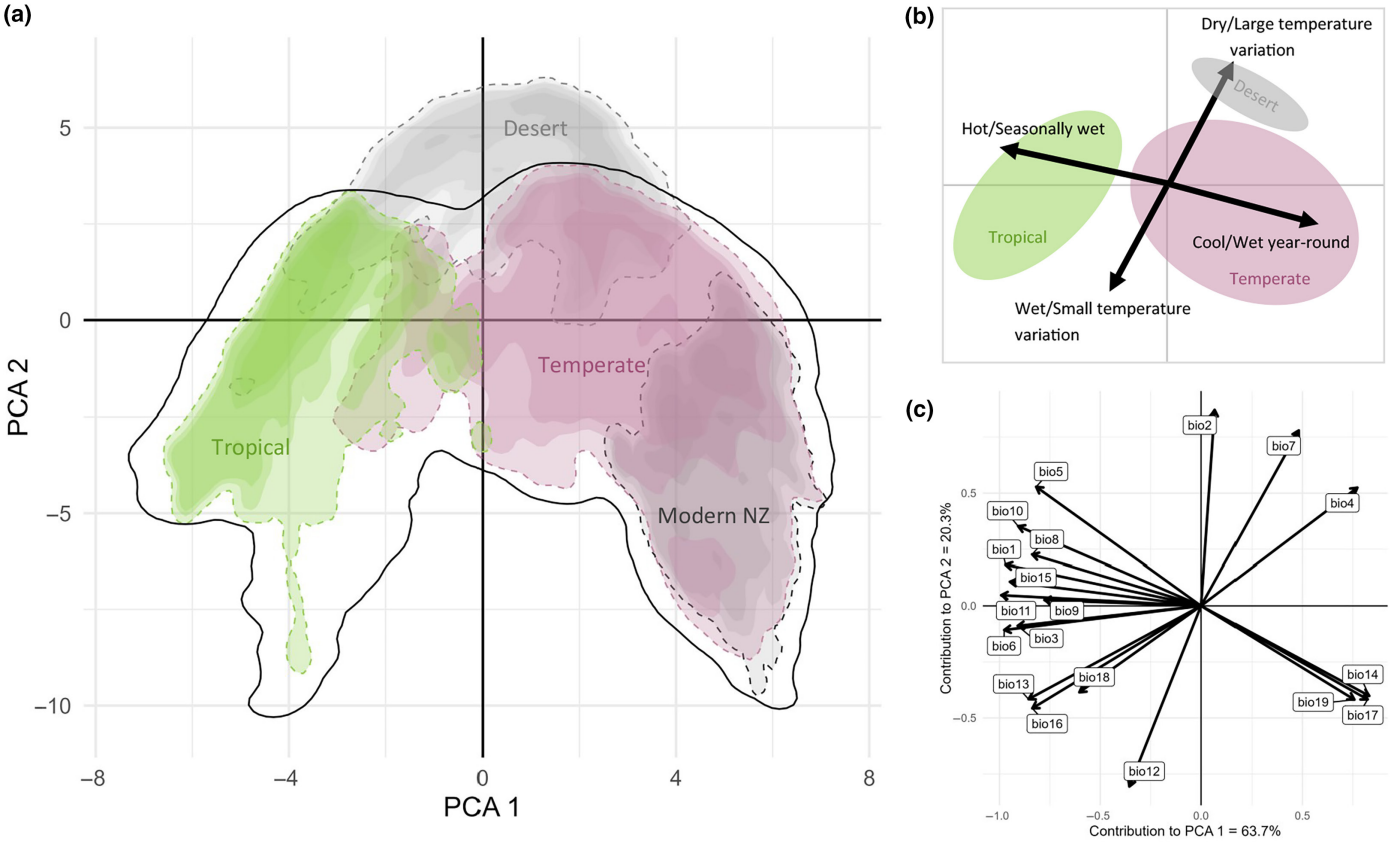
PCA results showing (a) the 99.99% density of tropical and temperate Australian climate points included in PCA construction (solid black outline) and the 99.95% density of climate space of different ecological areas: Tropical Australia (dashed green) and temperate Australia (dashed purple). Also shown are the distribution of areas not included in construction of the PCA: Desert Australia (dashed light gray) and modern New Zealand (dashed dark gray). (b) Description of main climate axes and where general climate zones occur. (c) Contribution of each bioclimatic variable along the first two PCA axes. Bioclimate variables that are included in the PCA are described in Section 2.

Australia currently contains climate regimes ranging from subtropical/tropical climates in the north, to temperate climates along portions western and eastern coasts, to dry desert climates in the interior (Figure 1). The temperate and tropical zones of Australia average annual temperature ranges from 5.0 to 29.1°C and annual precipitation from 318 to 3880 mm (Figure 1) which covers the entire range of New Zealand Cenozoic temperatures (>15 to 23°C, Table 1) and most of the New Zealand Cenozoic precipitation with the exception of extreme high precipitation in the Eocene and Pleistocene (1000 to 8000 mm, Table 1). The first axis in the temperate and tropical Australian climate PCA (PCA 1) explains 63.7% of the variation in the data and is strongly negatively correlated with temperature and precipitation seasonality variables (Figure 4). The second axis in the PCA (PCA 2) explains 20.3% of the variation in the data and is strongly positively correlated with annual precipitation and both daily and yearly temperature seasonality (Figure 4). Cenozoic New Zealand temperatures fall within the range of climates currently experienced in temperate and tropical Australia (Table 1). Cenozoic New Zealand precipitation is also well represented in the modern Australian climate space, however, precipitation extremes that are seen in Cenozoic New Zealand climate are missing from modern Australian climates (Figure 1 and Table 1). The modern New Zealand climate space largely overlaps with that of modern Australian climate space but is densely concentrated in the temperate and cool portions of Australia (Figure 4).

### 3.1 Niche volume

**FIGURE 5:**
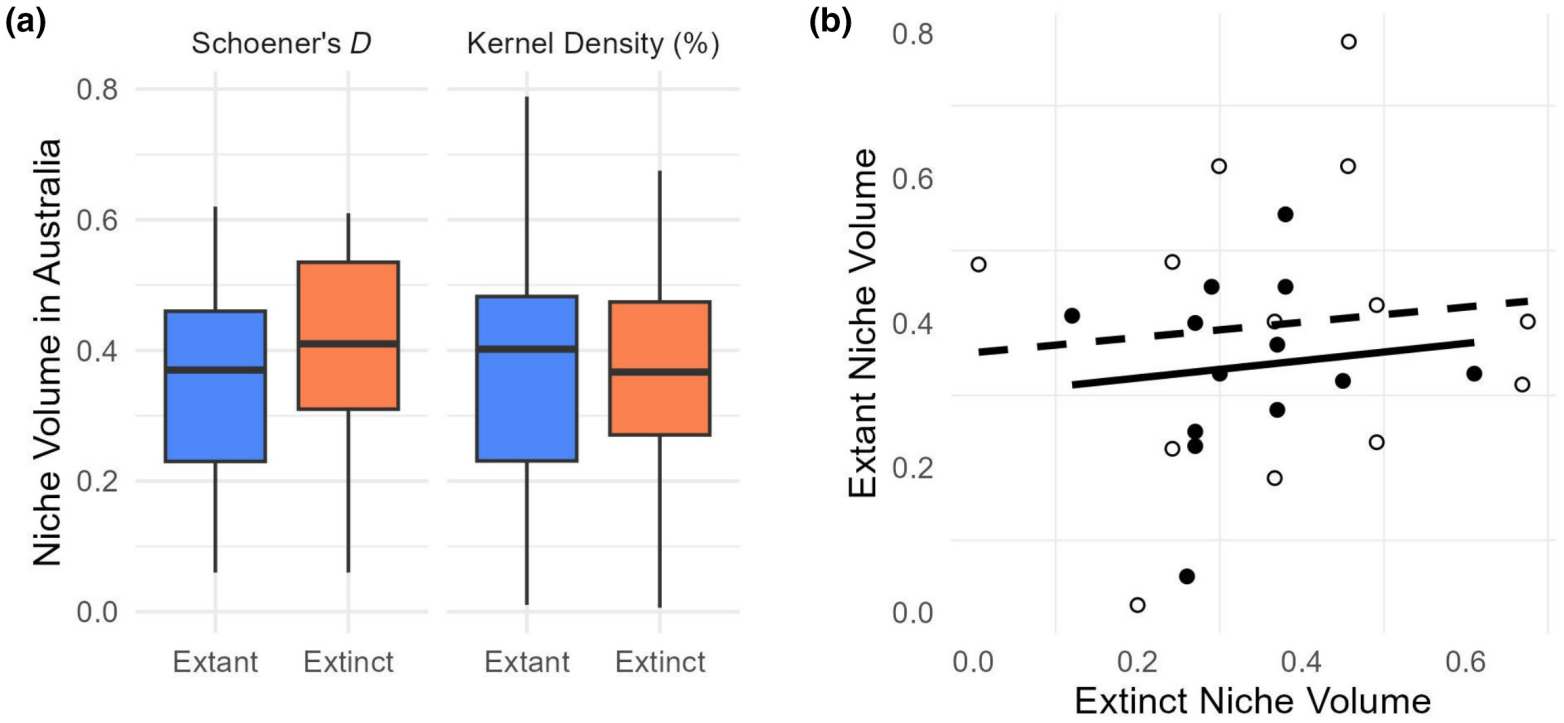
(a) Niche volume for extant (blue) and extinct (orange) genera in temperate and tropical Australia. Niche volume is calculated as the niche overlap (Schoener's *D*) and kernel density overlap of each genus' niche with the temperate and tropical Australian climate space. Comparison shows no significant difference in climatic niche volume between New Zealand extant and extinct genera in temperate and tropical Australia (Schoener's *D p* = .32, Kernel density *p* = .94, paired two‐tailed *t*‐test). (b) Comparison of niche volume between genus pairs, shows no correlation between individual genus pairs (Schoener's *D*, black points and black line, *p* = .72, *r* ^2^ = .01; Kernel density, open points and dashed line, *p* = .76, *r* ^2^ = .01).

We anticipated that if extinct New Zealand genera were extirpated by cooling Cenozoic climates in New Zealand, they would be restricted to the warmer climates of Australia and would have smaller climatic niches than extant genera in Australia. However, no significant difference was found in climatic niche volume between genus pairs. The climatic niche volume amongst genera varied considerably from low niche volume in Australian temperate and tropical climate space (*Corokia*, Schoener's *D* = 0.06, kernel density = 0.01) to moderate (*Euphorbia*, Schoener's *D* = 0.62, kernel density = 0.79). Although individual taxa vary widely, no difference is observed between the niche volume of New Zealand extant and extinct genera, with median niche volumes of Schoener's *D* values 0.37 and 0.41, respectively (*p* = .33 two‐tailed paired *t*‐test, Figure 5) and kernel density values of 0.38 and 0.38, respectively (*p* = .94 two‐tailed paired *t*‐test, Figure 5). Additionally, no correlation in niche volume is seen when comparing individual genus pairs (Schoener's *D p* = .72, *r* ^2^ = .01, kernel density *p* = .76, *r* ^2^ = .01; Figure 5).

### 3.2 Niche overlap and similarity

**FIGURE 6:**
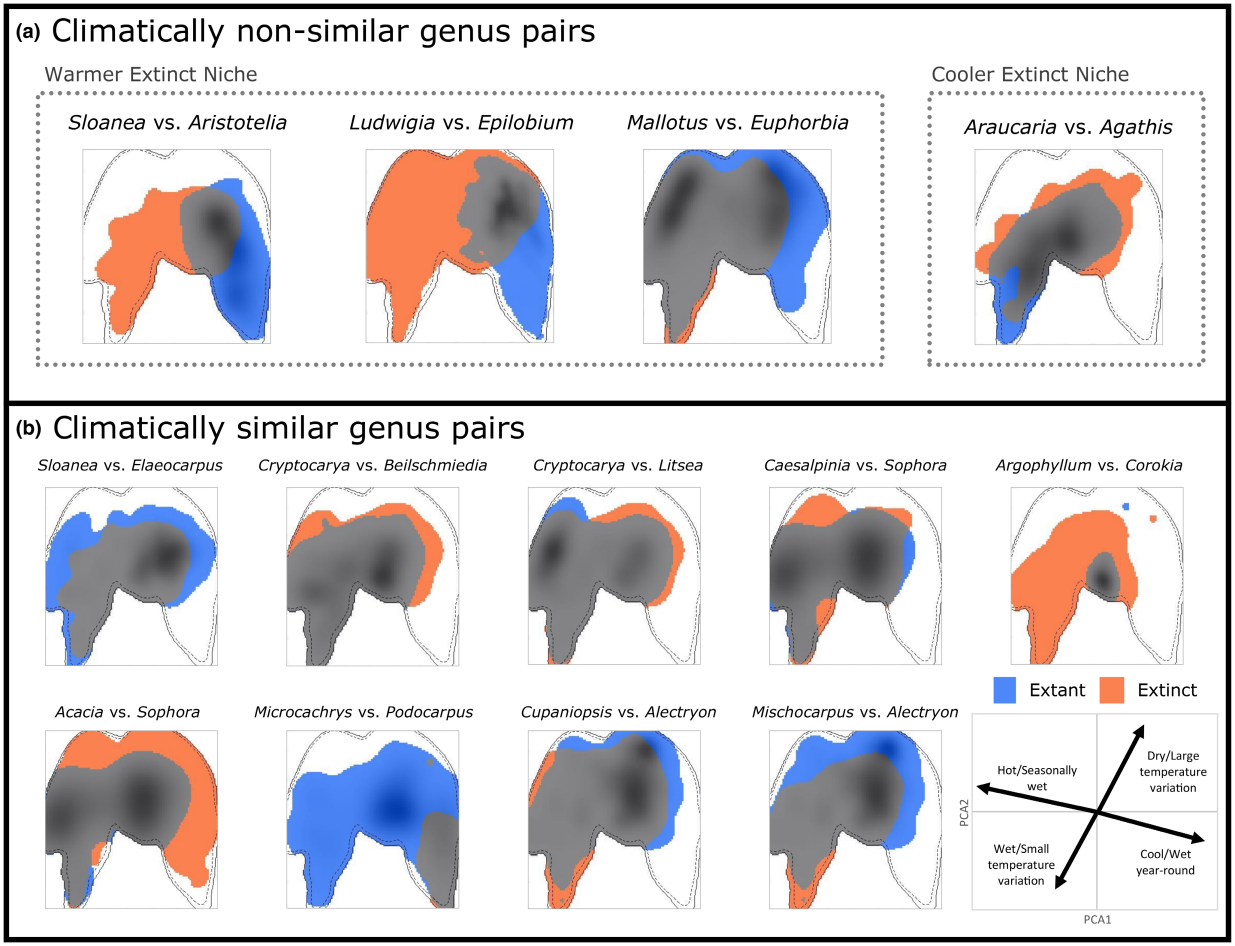
Realized climatic niche overlap for New Zealand extant (blue) and extinct (orange) genus pairs in Australian climate space for (a) climatically non‐similar pairs (similarity test *p* > .05 in both directions) and (b) climatically similar pairs. Outline of the tropical and temperate Australian climate space is depicted by the gray line.

A climatic niche legacy of extirpation by Cenozoic climate changes in New Zealand would be indicated by extinct climatic niches that are either not significantly similar to their extant niche counterpart (similarity test *p* > .05 in both directions) or are significantly different from their extant niche counterpart (difference test *p* < .05 in both directions). It is expected that if cooling Cenozoic climates were the extinction driver, extinct niches would occupy warmer portions of the modern Australian climate space than their extant counterparts. Comparison of the shape and location of extinct and extant genera's climatic niche within the Australian climate space shows that a majority of the genus pairs have significantly similar niches (69% or 9 out of 13 pairs; Figure 6, Table 2) and no niche pairs have significantly different niches (Table 2).

**FIGURE 7:**
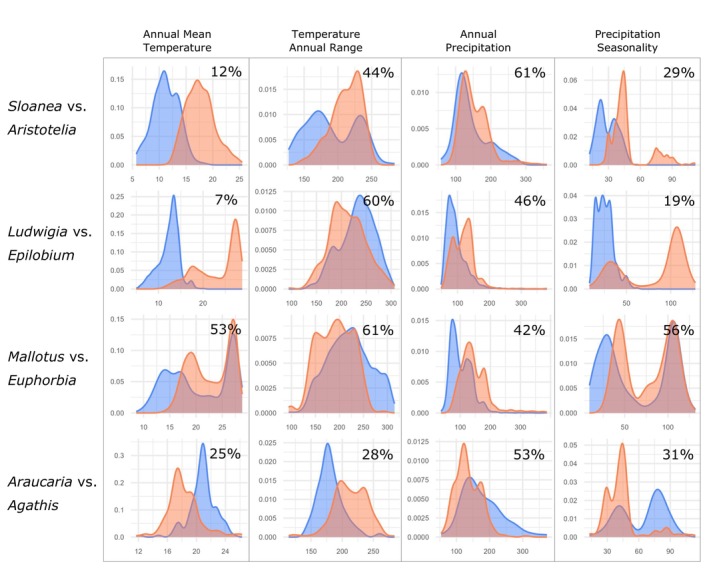
Kernel density overlap between taxa with significantly different climatic niches for key climate variables based on climatic niche of temperate and tropical Australia. Showing extinct niches in orange (*Sloenea*, *Ludwigia*, *Mallotus*, and *Araucaria*) and extant niches in blue (*Aristotelia*, *Epilobium*, *Euphorbia*, and *Agathis*). Percent kernel density overlap between each pair is shown within each graph.

Of the four pairs that are not statistically similar (similarity test *p* > .05 in both directions), three pairs show the expected warmer extinct niche pattern, *Sloanea* vs. *Aristotelia*, *Ludwigia* vs. *Epilobium* and *Mallotus* vs. *Euphorbia* (Figure 6). Both *Sloanea* vs. *Aristotelia* and *Ludwigia* vs. *Epilobium* also have very low niche overlap (Schoener's *D* = 0.09 and 0.06, respectively) while *Mallotus* vs. *Euphorbia* has comparatively high overlap (Schoener's *D* = 0.44; Figure 6, Table 2). This can be seen when comparing niche differences along individual climate gradients where both *Sloanea* vs. *Aristotelia* and *Ludwigia* vs. *Epilobium* have markedly different kernel density overlap along the mean annual temperature gradient (12% and 7%, respectively) with extinct genera occupying warmer areas. The kernel density overlap of precipitation seasonality for the two pairs is slightly more similar (29% and 19%, respectively) with extinct genera in areas with larger precipitation seasonality (Figure 7). This suggests that the extinct genera *Sloanea* and *Ludwigia* occupy climates that are warmer with more distinct wet and dry seasons than their extant counterparts *Aristotelia* and *Epilobium*. These patterns are also seen with *Mallotus* vs. *Euphorbia* but with greater kernel density overlap values than the other two pairs (Figure 7). Kernel density overlap in temperature annual range and annual precipitation are large and likely not driving the differences between these genera (Figure 7).

The final genus pair that is not similar, *Araucaria* vs. *Agathis*, shows the opposite trend with the extinct genus occupying areas that are cooler with less precipitation seasonality (Schoener's *D* = 0.33; Figure 6 and Table 2). Comparison of individual climate gradients show low overlap with the extinct genus found in cooler areas that have consistent year‐round precipitation but also have slightly more seasonal temperature variation suggesting that the extinct *Araucaria* occupies more temperate climates than its extant counterpart *Agathis* (Figures 6 and 7).

## 4 DISCUSSION

### 4.1 Potential climate legacies of extinction events

Through analysis of climatic niche similarities and differences within genus pairs, we found that four extinct taxa show potential climate legacies with non‐similar niches to their extant counterpart in the similarity test; however, this did not translate to the difference test which showed no pairs had significantly different niches. Three extinct taxa have warmer niches than their closely related extant taxa pair (*Sloanea*, *Ludwigia*, and *Mallotus*; Figures 6 and 7) which provides support for the importance of climate cooling events in the extinction of genera from New Zealand during the Cenozoic. The last occurrence of two taxa with warmer niches (*Sloanea* and *Mallotus*) coincided with two periods of pronounced climate cooling in New Zealand during the last 30 million years (Prebble et al., 2017). Although the last occurrence of *Sloanea* (15–18 MYA) occurs before the Middle Miocene Climatic Optimum (MMCO, ~14 MYA), the extinction date of *Sloanea* is particularly susceptible to the Signor–Lipps effect, which is the low likelihood that the fossil of the last individual is recovered therefore the true extinction date would occur after the date of the last fossil recovered (Signor III & Lipps, 1982). This is due to macrofossils recovered from abundant early‐middle Miocene lacustrine sediments of the Manuherikia Group (Pole, 1993, 2007), whereas fossil‐bearing strata of the late Miocene are much rarer. Therefore, the extinction date of *Sloanea* may have occurred in the late Miocene during climate cooling (Prebble et al., 2017) but be undetectable in the fossil record. The late Miocene cooling after the MMCO was particularly pronounced, with minimum temperatures cooling by approximately 6°C, even while maximum temperatures remained stable at approximately 20°C (Prebble et al., 2017). This change in temperature may have had a pronounced impact on *Sloanea* whose average mean annual temperature is 6.4°C warmer than its extant counterpart *Aristotelia* (Figure 7). The last occurrence of *Mallotus* (2–3 MYA) coincides with a second period of pronounced climatic cooling at the Pliocene‐Pleistocene transition when both maximum and minimum temperatures in New Zealand decreased by 1.5 and 6°C, respectively (Prebble et al., 2017). This is consistent with the warmer climatic niche of *Mallotus*, which occupies areas that are on average 2.2°C warmer than its extant counterpart *Euphorbia*.

The third taxa with a warmer niche, *Ludwigia*, has an extinction in New Zealand (23–25 MYA) that coincides with the Oligocene‐Miocene transition (~23.7–22.7 MYA), which is a period of regional cooling associated expansion of Antarctic glaciation (Liebrand et al., 2017). During a period of 1 million years, ice sheets expanded to approximately the size of the modern East Antarctic Ice Sheet and subsequently disintegrated to the pre‐transition volume (Liebrand et al., 2017). This suggests a period of rapid regional cooling that is consistent with extinction of *Ludwigia*, which occupies areas that are on average 10.8°C warmer than its extant counterpart *Epilobium* (Figure 7).

Adaptations to climate cooling involve multiple physiological processes and, in thermophilic species, may limit growth and disrupt species interactions (Cahill et al., 2013). In Europe, most widespread temperate taxa have a higher degree of cold‐tolerance than regionally extinct or relic species due to environmental filtering during the cool Pliocene and Pleistocene (Svenning, 2003). This is also seen in Japan and Tasmania where many cold‐intolerant plant species went extinct in response to early Pleistocene cooling (Huang et al., 2018; Jordan, 1997). However, in New Zealand many temperate tree taxa still share functional traits typically associated with tropical species, such as thin bark and gender dimorphism (McGlone et al., 2016). Additionally, warm adapted taxa, such as *Ludwigia*, have recently been introduced to New Zealand and in other introduced regions *Ludwigia* is predicted to expand its range in response to increased temperatures from anthropogenic climate change (Gillard et al., 2017).

*Araucaria* occupies areas in Australia that are cooler and with less seasonal variation in precipitation and temperature than its New Zealand extant counterpart *Agathis* but has similar overall precipitation (Figures 6 and 7). The last occurrence of *Araucaria* (2–3 MYA) occurs during a period of climate cooling during the Pliocene‐Pleistocene transition. This was also a period of increased total and more variable precipitation in New Zealand (Prebble et al., 2017) which may have been a determinant of *Araucaria* extinction in New Zealand due to *Araucaria* occupying areas with lower precipitation seasonality than its extant counterpart *Agathis* (Figure 7). *Araucaria* is also no longer present in southern Australia although fossil evidence indicates it was common in the past (Hill, 1990) suggesting that *Araucaria* migrated northward to more favorable temperate and subtropical climates in Australia. However, migration may not have been possible in New Zealand, due to the limited northern extent of the landmass. The temperature trends during the Pliocene‐Pleistocene transition would suggest that *Araucaria* would outcompete its extant counterpart during this period, with an average mean annual temperature of 2.5°C cooler than the extant *Agathis*. This effect is evident in South America where *Araucaria* forests have decreased their range since the LGM in response to warming and are predicted to suffer further range restrictions due to anthropogenic climate change (Bergamin et al., 2019). However, pronounced glacial cycles that developed during the Pliocene‐Pleistocene transition (Mildenhall et al., 2004; Naish et al., 1997) may negatively impact taxa that occupy areas with low seasonal variation such as *Araucaria*. For example, the disappearance of Miocene seasonal climates has been suggested to have led to the loss of other flora species in New Zealand, such as the deciduous Nothofagaceae species which still occupy highly seasonal climates in South America (Reichgelt et al., 2022).

### 4.2 Extinction events lacking climatic niche legacies

Although significant climatic niche legacies are not evident in the results of most taxa investigated here, many went extinct during the Miocene, Pliocene, and Pleistocene epochs (*Acacia*, *Araucaria*, *Argophyllum*, *Cryptocarya*, *Cupaniopsis*, *Ludwigia*, *Mallotus*, *Microcachrys*, *Mischocarpus*, and *Sloanea*) associated with pronounced long‐term, and sometimes more accelerated climate cooling or rapid climate fluctuations. Only one taxa extinction occurred during the relatively climatically stable Oligocene (*Caesalpinia*). The lack of a consistent discriminating climate indicator for these genus pairs suggests that extinction in New Zealand may be explained by two mechanisms: (i) niches are conserved, suggesting that the mechanism of extinction was not climate change directly but either an indirect effect impacting taxa environmental tolerance or the result of a non‐climatic factor, such as habitat loss, or (ii) the climatic niches have evolved to occupy overlapping climates in Australia and any climate legacies that may have existed at the time of extinction have been lost.

The degree to which climatic niches are conserved through time may impact our ability to detect climatic niche legacies, with extinct taxa that underwent high levels of niche evolution potentially obscuring detection of climatic niche legacies. However, niche conservation during periods of large climatic changes has been reported in various taxa. A study comparing sister taxa in Europe, North America, and East Asia found that cool‐temperate tree genera have conserved their niches since the Pliocene (Svenning, 2003). Niche conservation has also been witnessed in *Fagus* between the North American and European species which were geographically separated during the breakup of Laurasia (Huntley et al., 1989). The climatic niche of *Laurus* (Lauraceae) in Europe has also remained conserved since the middle Pliocene (3 MYA), consistently occupying areas that are warm and wet with little seasonal variation (Rodríguez‐Sánchez & Arroyo, 2008). The climatic niche of the tree species *Carpinus betulus* and *Picea abies* in Europe have been conserved since the middle Holocene despite large changes in environmental conditions (Pearman, Randin, et al., 2008). Assuming niche conservatism within taxa suggests that the lack of climate niche legacy is the result of the first proposed mechanism – that niches are conserved and that either non‐climatic or indirect climatic factors were the drivers of extinction.

Non‐climatic factors such as habitat loss may have played a role in the *Caesalpinia* whose extinction age coincides with the period of maximum marine transgression in New Zealand (28 MYA) and associated habitat loss from rising sea levels (Cooper & Cooper, 1995). Extinction caused habitat loss from sea‐level rise was also seen during the Pleistocene in Bermuda when a large reduction in land area was followed by the extinction of many bird species (Olson & Wingate, 2000). Marine transgression also caused New Zealand to split into a series of small islands (Cooper & Cooper, 1995) which may have increased the extinction rate during this period due to decreased land area, genetic isolation, or increased competition for limited resources (Rosenzweig, 1995). However, there is little evidence that changes in land area is a contributor to extinctions in New Zealand, as the maximum marine transgression of the Oligocene witnessed markedly few extinctions (Lee et al., 2001). Other potential non‐climatic extinction mechanisms could include geologic changes in the mid‐ to late‐Cenozoic resulting in the loss of soil types, such as deep, highly weathered nutrient poor soils, that developed during the early Cenozoic (McGlone et al., 2016).

Climate changes may have also had indirect impacts on genera's ability to survive through changes in species interactions or disturbance regimes that are not reflected in the current realized niche (Cahill et al., 2013). Families' dependent on insect pollination, for example, may have been susceptible to changes in abundance of sub‐tropical insects. Conran et al. (2014) found that of the 30 genera in 23 families identified at the 23 million‐year‐old Foulden Maar deposit in New Zealand, all had retained their reproductive niches, suggesting co‐extinction could play a role in plant extinctions. Additionally, it is suggested that after the maximum marine transgression of the late Oligocene, New Zealand may have contained many relatively vacant niches resulting in a high level of long‐distance dispersal and colonization from Australia, including putative evidence for *Eucalyptus* (Lee et al., 2012; Pole, 1993), which appeared in New Zealand during the Miocene (Lee et al., 2016; McGlone et al., 2001). These colonization events may have introduced competitors, leading to the extinction of genera in the early to middle Miocene (extinction of *Sloanea*, *Cryptocarya*, and *Argophyllum*). However, further studies are needed to determine if these events resulted in extinction. Additionally, changes in disturbance regimes may have influenced taxa survival. There is evidence that fire was more prevalent in the mid‐Miocene when fire adapted genera, such as *Acacia*, were prevalent in New Zealand, but subsequently went extinct (McGlone et al., 2016). A gradual change to less fire‐prone vegetation, as rainforests expanded, may have resulted in the extinction of pyrophilic genera, as is observed in southeast Australia today (Read & Hill, 1985).

Although many taxa conserve their climatic niches through time, some taxa respond to environmental changes through niche evolution. Studies that aim to determine niche conservatism and divergence within families show evidence of general niche conservatism but with occasional taxa undergoing niche divergence at the species level (Bystriakova et al., 2011). Tracing niche evolution within genera shows that speciation can cause niche divergence. This is seen in the *Chrysanthemum zawadskii* species complex in which niche evolution occurred with speciation as they migrated into new geographic areas (Lu et al., 2023). This can also be seen within a single species, such as the European *Juniperus communis*, whose niche has diverged from its middle Holocene niche, likely due to changes in habitat and competition dynamics rather than climate tolerance (Pearman, Randin, et al., 2008). Enhanced evolutionary pressure from competition can also be seen in other aspects of species' traits, such as stature, with more trait divergence amongst species with competitors than those without (Burns, 2022). Studies inspecting evolutionary responses to competition and habitat availability have largely been focused on at the species level (e.g. Ackerly et al., 2006; Emery et al., 2012; Lu et al., 2023) and it remains unclear if the same dynamics occur at higher taxonomic levels.

## 5 CONCLUSION

Using climatic niche comparisons, we show no universal climatic niche legacy in the Australian modern assemblage of New Zealand extinct and extant genera. We do provide some evidence that cold intolerance was potentially responsible for three extinctions during Cenozoic cooling events. This supports previous hypotheses about the importance of climate filtering in the composition of modern flora (Lee et al., 2001). In addition to temperature, differences in precipitation seasonality were found to be important in one investigated genus pair. However, the majority of the taxa examined here did not show detectable climatic niche legacies of extinction. This suggests that either climate had an indirect role in extinction event, such as through changes in species interactions, or that changes in non‐climatic mechanisms, such as geologic or tectonic changes, prevented persistence in New Zealand. Our results suggest that the mechanisms of Cenozoic extinctions of New Zealand plant genera may be more complex than taxa reaching environmental tolerance limits from cooling climates and more research is needed to determine New Zealand Cenozoic extinction drivers. Recommendations for future work include: (i) analyzing the relationship between phylogenetic similarity and niche similarity at various taxonomic levels to better understand how taxa evolution impacts current environmental niches; (ii) conducting niche comparison studies within genera to determine the level of niche conservatism, especially for those with distributions spanning the global temperate to tropical climate space, such as South America; (iii) analyzing individual genus‐specific species interactions to determine how prevalent obligate mutualisms are amongst extinct genera and how susceptible taxa with symbiosis may have been to cooling climates.

## AUTHOR CONTRIBUTIONS

**Nora Schlenker:** Data curation (lead); formal analysis (lead); investigation (lead); methodology (equal); writing – original draft (lead); writing – review and editing (equal). **William G. Lee:** Conceptualization (equal); resources (equal); writing – review and editing (equal). **Tammo Reichgelt:** Conceptualization (equal); resources (equal); writing – review and editing (equal). **Ralf Ohlemüller:** Conceptualization (equal); methodology (equal); resources (equal); writing – original draft (supporting); writing – review and editing (equal).

## CONFLICT OF INTEREST STATEMENT

The authors declare no known competing interests relating to the work presented.

## Data Availability

The data that support the findings of this study are openly available from GBIF (2023) with specific data references included in Appendix 1. Code for all analyses is openly available at https://zenodo.org/doi/10.5281/zenodo.12534920.

## APPENDIX 1 Full references for species distribution data downloads and summary of data points

### 1.1

GBIF.org 29 April 2019a. GBIF Occurrence Download https://doi.org/10.15468/dl.3hry6r.

GBIF.org 29 April 2019b. GBIF Occurrence Download https://doi.org/10.15468/dl.m71cud.

GBIF.org 29 April 2019c. GBIF Occurrence Download https://doi.org/10.15468/dl.mrehyy.

GBIF.org 29 April 2019d. GBIF Occurrence Download https://doi.org/10.15468/dl.nlwct2.

GBIF.org 29 April 2019e. GBIF Occurrence Download https://doi.org/10.15468/dl.pljxpc.

GBIF.org 29 April 2019f. GBIF Occurrence Download https://doi.org/10.15468/dl.pqi1zj.

GBIF.org 29 April 2019g. GBIF Occurrence Download https://doi.org/10.15468/dl.t472lw.

GBIF.org 29 April 2019h. GBIF Occurrence Download https://doi.org/10.15468/dl.ukoj92.

GBIF.org 29 April 2019i. GBIF Occurrence Download https://doi.org/10.15468/dl.xhh2os.

**Table:**

Family	Genera	GBIF occurrence points	Climate grid cells	Number of species
Araucariaceae	Araucaria	1645	644	6
	Agathis	808	154	4
Argophyllaceae	Argophyllum	634	154	4
	Corokia	252	28	1
Elaeocarpaceae	Sloanea	2766	762	4
	Elaeocarpus	16,789	3408	26
	Aristotelia	347	217	3
Euphorbiaceae	Mallotus	5969	1419	15
	Euphorbia	13,175	3706	79
Fabaceae	Acacia	351,665	22,421	854
	Caesalpinia	1047	203	16
	Sophora	588	62	7
Lauraceae	Cryptocarya	23,078	2644	52
	Beilschmiedia	3244	505	12
	Litsea	5406	1471	13
Onagraceae	Ludwigia	3646	1603	10
	Epilobium	9599	4256	22
Podocarpaceae	Microcachrys	137	43	1
	Podocarpus	2534	502	12
Sapindaceae	Cupaniopsis	5545	1326	15
	Mischocarpus	2588	559	11
	Alectryon	5806	1303	14

## APPENDIX 2 Presumed extinction dates with associated references.

### 2.1

**Table:**

Family	Genera	Last occurrence (MYA)	References
Araucariaceae	Araucaria	2–3	Pole (2008a)
Argophyllaceae	Argophyllum	15–18	Pole (2008b)
Elaeocarpaceae	Sloanea	15–18	Pole (1993b), Conran et al. (2014)
Euphorbiaceae	Mallotus	2–3	Lee et al. (2010), Conran, Lee, and Reichgelt (2016)
Fabaceae	Acacia	1	Mildenhall (1980), Mildenhall (1989)
	Caesalpinia	28	Pocknall (1982)
Lauraceae	Cryptocarya	15–18	Pole (2007)
Onagraceae	Ludwigia	23–25	Pocknall (1991), Bannister et al. (2005)
Podocarpaceae	Microcachrys	1	Jordan et al. (2010), Mildenhall (1980)
Sapindaceae	Cupaniopsis	2–3	Mildenhall (1980), Lee et al. (2016)
	Mischocarpus	2–3	Mildenhall et al. (1992)
